# Neural Correlates of Consumer Buying Motivations: A 7T functional Magnetic Resonance Imaging (fMRI) Study

**DOI:** 10.3389/fnins.2017.00512

**Published:** 2017-09-14

**Authors:** Adam M. Goodman, Yun Wang, Wi-Suk Kwon, Sang-Eun Byun, Jeffrey S. Katz, Gopikrishna Deshpande

**Affiliations:** ^1^Department of Psychology, Auburn University Auburn, AL, United States; ^2^Department of Psychology, University of Alabama at Birmingham Birmingham, AL, United States; ^3^Department of Electrical and Computer Engineering, AU MRI Research Center, Auburn University Auburn, AL, United States; ^4^Department of Consumer and Design Sciences, Auburn University Auburn, AL, United States; ^5^Department of Retailing, University of South Carolina Columbia, SC, United States; ^6^Alabama Advanced Imaging Consortium, Auburn University and University of Alabama at Birmingham Birmingham, AL, United States

**Keywords:** consumer, motivation, decision, fmri, prefrontal cortex

## Abstract

Consumer buying motivations can be distinguished into three categories: functional, experiential, or symbolic motivations (Keller, 1993). Although prior neuroimaging studies have examined the neural substrates which enable these motivations, direct comparisons between these three types of consumer motivations have yet to be made. In the current study, we used 7 Tesla (7T) functional magnetic resonance imaging (fMRI) to assess the neural correlates of each motivation by instructing participants to view common consumer goods while emphasizing either functional, experiential, or symbolic values of these products. The results demonstrated mostly consistent activations between symbolic and experiential motivations. Although, these motivations differed in that symbolic motivation was associated with medial frontal gyrus (MFG) activation, whereas experiential motivation was associated with posterior cingulate cortex (PCC) activation. Functional motivation was associated with dorsolateral prefrontal cortex (DLPFC) activation, as compared to other motivations. These findings provide a neural basis for how symbolic and experiential motivations may be similar, yet different in subtle ways. Furthermore, the dissociation of functional motivation within the DLPFC supports the notion that this motivation relies on executive function processes relatively more than hedonic motivation. These findings provide a better understanding of the underlying neural functioning which may contribute to poor self-control choices.

## Introduction

Motivation can be broadly defined as an internal impetus to act and carry out behaviors which can vary in both intensity and type of valuation (Ryan and Deci, 2000). The predominant model of consumer buying motivations has identified functional, experiential, and symbolic types of values as fundamental needs (Keller, 1993). According to this theoretical understanding of consumer motivations, information processing contributing to a purchase decision differs for each motivation. When individuals are motivated by functional needs, their decision making is primarily determined by instrumental benefits (e.g., financial savings, quality, or security); whereas when their motivation is experiential, their decision is determined by emotional or hedonic benefits such as the fun and excitement the product can provide (Dhar and Wertenbroch, 2000). An individual's purchase decision can also be motivated by symbolic needs based on social cognition where they seek products that provide social benefits such as conveying their taste and status or enhancing their self-image in a social setting (Keller, 1993; Verplanken and Sato, 2011). Prioritization of self-directed functional, experiential, and symbolic valuation of products is a reliable predictor of purchasing behavior (Kim et al., 2002). Dissociations of functional, experiential, and symbolic motivations have been well-documented in behavioral studies and shown to influence purchasing behavior. However, new knowledge about the neural bases of functional, experiential, and symbolic valuations will provide increased understanding of the mechanisms involved in consumer decisions and differences in information processing between consumer motivations.

Among the approaches to understanding the distributed neural functioning which enables motivational values, consumer choice methods are ideal. As an increasingly popular topic in neuroimaging literature, consumer choice-making studies have extended the understanding of how people interact and behave in a contemporary environment (Lee et al., 2007). Thus, the implications of these studies expand well beyond commercial aims and relate broadly to examinations of consumer choice and decision-making. One traditional area of consumer research with limited utilization of neuroimaging methods has been investigations of motivational states on consumer choices. Although support for the internal validity of the functional, experiential, and symbolic motivations has been demonstrated behaviorally, to date, limited studies have directly examined these consumer buying motivations using functional neuroimaging to validate the proposed differences in information processing across various motivations (Erk et al., 2002; McClure et al., 2004; Tsai et al., 2006; Levy et al., 2011).

Erk et al. (2002) examined functional and symbolic consumer motivations using fMRI during an image rating task. They found that artificial cultural objects (i.e., luxury and sport cars) associated with wealth and social dominance elicit activation in reward-related brain areas (i.e., right ventral striatum, left anterior cingulate and bilateral prefrontal cortex). Alternatively, practical and economical objects (i.e., small cars) were associated with deactivations among these regions (Erk et al., 2002). In a similar study of functional and symbolic motivations, Schaefer and Rotte (2007) assessed the neural basis for different categories of culturally based brands affecting people's purchasing decisions. The findings revealed that the medial prefrontal cortex (MPFC) and precuneus demonstrated greater activation when comparing symbolic motivation (i.e., sports and luxury car brands) to functional motivation (i.e., unfamiliar product labels or value brands). This finding led the authors to assert that the perception of brands that are related to high or low social dominance modulated purchasing decisions via greater activations in the MPFC and precuneus.

Prior neuroimaging findings involving comparisons of functional and symbolic motivations are largely consistent with the theoretical understanding of these valuations. The MPFC has been related to self-reflection and self-relevant processing (Johnson et al., 2002; Ochsner et al., 2004). Additionally, both the anterior medial cortex (medial frontal gyrus and anterior cingulate cortex) and posterior medial cortex (PCC and/or precuneus) have been implicated in self-referential processing (Ochsner et al., 2004; Vogt and Laureys, 2005). Johnson et al. (2006) dissociated MPFC and PCC activity during self-reflection, positing that the MPFC is associated with instrumental or agentic self-reflection, whereas the PCC is associated with experiential self-reflection. Medial areas of the prefrontal cortex (PFC) have traditionally been implicated in affective/motivational systems, whereas lateral areas tend to be more involved in sensory/motor processing. Accordingly, O'Reilly's (2010) model has proposed a functional division within the cognitive control network of the PFC in which the lateral PFC underlies goal-oriented behavior guided by non-arousing, neutral information (*cold processing)*, whereas the medial PFC underlies goal-oriented behavior guided by arousing and pleasant information (*hot processing*).

One limitation of prior neuroimaging studies of consumer motivations involves comparing only functional and symbolic valuations. Keller (1993) identifies fundamental values as functional, experiential, and symbolic. Thus, it remains unclear whether self-referential neural activity is associated with both symbolic and experiential, but not with functional motivations. Furthermore, self-referential valuations may be associated with inward or outward reflection (Johnson et al., 2006). Accordingly, investigations to dissociate the neural circuitry between experiential and symbolic motivations, and how these uniquely differ from functional motivations have remained elusive. By comparing each of Keller's (1993) three motivations using an instructed valuation task, we hypothesized that dissociations within the lateral to medial PFC would emerge with respect to functional, experiential, and symbolic types of consumer motivations.

The current study aimed to test this hypothesis in the context of a consumer decision task during blood-oxygen-level-dependent (BOLD) fMRI data acquisitions. Based on the prior literature discussed above, it was hypothesized that task-related activations during the functional condition should be associated with greater activity in cognitive-control related regions previously implicated in studies involving a response to instrumental benefits, in particular, the dorsolateral prefrontal cortex (DLPFC). Task-related activations during the experiential condition should lead to greater activity in regions including the PCC associated with inward-directed self-reflection, such as self-emotional or self-hedonic benefit. Task-related activations during the symbolic condition should lead to greater activity in regions including the MPFC implicated in the outward-directed self-reflection process, such as social benefits.

## Methods

### Participants

Participants were recruited following an initial interview in which volunteers self-reported being right-handed and having normal or corrected-to-normal vision. All participants provided written informed consent prior to any training or image acquisition and were financially compensated for their time. Ten volunteers (age range = 19–24 years; five females) served as experimental subjects. A power analysis technique (Mumford and Nichols, 2008) was used to decide the sample size. Accordingly, we based the effect sizes on a subset of participants (*n* = 5) and our regions of interests (MPFC, PCC and DLPFC, defined from Automated Anatomical Labeling atlas). The power curve revealed that 10 subjects were sufficient to achieve 75% calculation power for group fMRI experiments (See Supplementary Figure 1). All experimental methods and procedures were approved by the Auburn University Institutional Review Board.

### Stimuli

Eighteen generic products were selected based on the results from a series of pilot studies administered to Auburn University undergraduates. Through the first pilot survey (*n* = 135), a pool of 152 products appealing to college-aged consumers were identified, among which 34 products were then selected to be gender-neutral and non-seasonal by a panel of experts (*n* = 7) consisting of three and two faculty members from consumer sciences and psychology, respectively, and two graduate students from consumer sciences. The second pilot survey (*n* = 102) established 18 among the 34 products to represent varying levels of expensiveness (ranging from “not expensive at all” to “very expensive”) and buying and promotion frequencies (ranging from “once every few years” to “always”) to college students. Four of these generic products (i.e., car, pasta sauce, sunglasses, and tablet computer) were selected to serve as training stimuli, which were used in practice versions of the task (to be performed outside the scanner) designed to be similar to the actual task. The remaining 14 products (bikes, blu-ray players, books, cellphones, cellphone cases, drinks, DVDs, laptop computers, mugs, plane tickets, shower curtains, towels, TVs, and workout apparel) were presented in pictorial stimuli which visually and verbally depicted these consumer goods in scenarios representing either a functional, experiential, or symbolic motivational condition.

**Figure 1:**
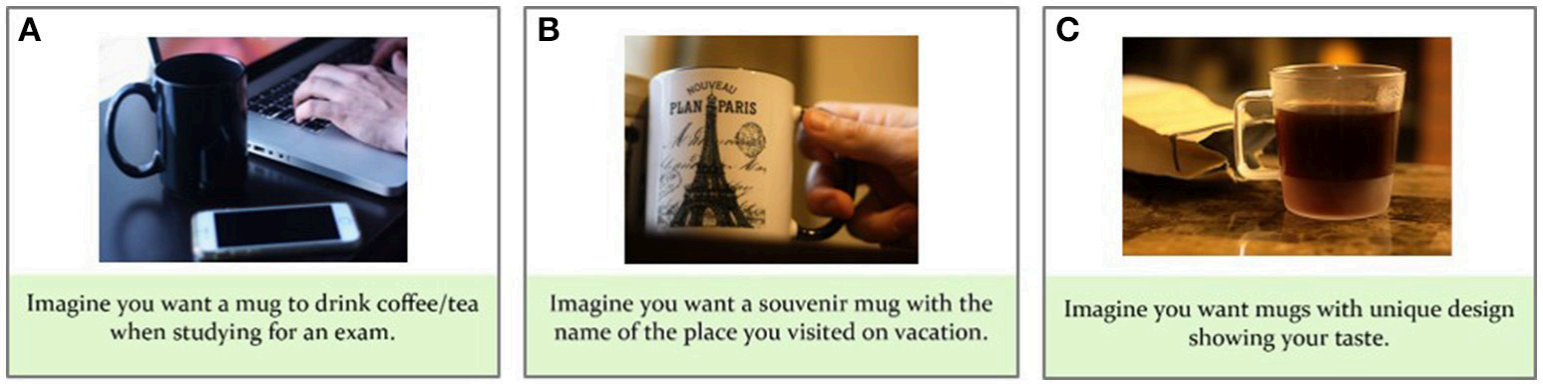
Example stimuli. Examples of three motivational categories for products (e.g., mugs) depicted in pictorial stimuli with instructed buying motivations for the product. **(A)** Functional, **(B)** experiential, and **(C)** symbolic motivation. Note that none of the example images are part of the experimental stimulus material used in the current study. The copyright lies with the authors, and no permission was required for the reproduction of these images.

For each product, three scenarios were created per motivational condition so that they contained varying visual and verbal descriptions of the same motivational condition. Therefore, nine stimuli were generated by nesting the three motivational conditions and the three visual/verbal scenarios for each of the 14 generic consumer goods (see Figure 1), resulting in a total of 126 buying scenarios. The 126 scenarios were subjected to a behavioral pretest with a sample of 274 Auburn University undergraduates. To prevent disturbance effects from subjects' fatigue, each pretest subject evaluated only a partial set among the 126 scenarios assigned to them according to a mixed design. First, the 14 products were grouped into two sets of seven products. The subjects were randomly assigned to one of the two product sets. Then, they were shown all nine motivational condition scenarios of each assigned product. Each scenario was rated on one of the following three motivational likelihood questions, randomly assigned: “If you shop for [product name] for the above occasion, how likely are you to consider” (1) “its functional and practical aspects” (i.e., functional value), (2) “the fun and excitement it can offer” (i.e., experiential value), or (3) “whether it can tell something about yourself” (i.e., symbolic value). The questions were answered using a 5-point Likert scale (1 = very unlikely, 5 = very likely), and the scenario presentation order was counterbalanced across subjects. This mixed design led to six stimulus blocks (see Table 1), each of which was individually analyzed employing repeated measures ANOVA for a three-way Product (7) × Motivation Scenario (3) × Motivational Likelihood Question (3) design. Motivational Likelihood Question was a between-subjects factor, whereas Product and Motivation Scenario were within-subjects factors. The results revealed significant (*p* < 0.001) Motivation Scenario × Motivational Likelihood Question interaction effects for all six stimulus blocks, confirming that the subjects were most likely to consider the respective product values that matched the motivational scenarios of the stimuli (see Table 1).

**Table 1:** Results from the behavioral pretest of the stimuli.

**Stimulus block^a^**	**Motivation scenario**	**Likelihood to consider^b^**			***F*(*df1, df2*)^c^**	***p***
		**Functional value**	**Experiential value**	**Symbolic value**		
1	Functional	4.58	3.09	3.32	77.34 (4, 266)	<0.001
	Experiential	3.44	4.09	3.63		
	Symbolic	3.40	3.75	4.05		
2	Functional	4.21	2.61	2.97	71.52 (4, 266)	<0.001
	Experiential	3.63	3.98	3.71		
	Symbolic	3.47	3.52	4.04		
3	Functional	4.46	2.54	2.48	102.47 (4, 266)	<0.001
	Experiential	3.46	3.91	3.61		
	Symbolic	3.05	3.60	4.19		
4	Functional	4.30	2.57	2.47	73.23 (4, 270)	<0.001
	Experiential	3.43	4.07	3.39		
	Symbolic	3.23	3.60	3.70		
5	Functional	4.25	2.97	2.88	71.31 (4, 270)	<0.001
	Experiential	3.30	4.01	3.26		
	Symbolic	3.08	3.46	3.85		
6	Functional	4.25	2.67	2.61	90.35 (4, 270)	<0.001
	Experiential	3.24	4.18	3.33		
	Symbolic	2.87	3.26	3.88		

a *Stimulus blocks 1 through 3 used seven products (laptop computers, mugs, plane tickets, shower curtains, towels, cell phones, and workout apparel), whereas stimulus blocks 4 through 6 used the remaining seven products. Each stimulus block contained only one of the three scenarios tested for each of its respective product × motivation cells*.

b *The reported numbers are means*.

c *The test statistics reported are for the two-way Motivation Scenario (3) × Motivational Likelihood Question (3) interaction*.

### Task design

**Figure 2:**
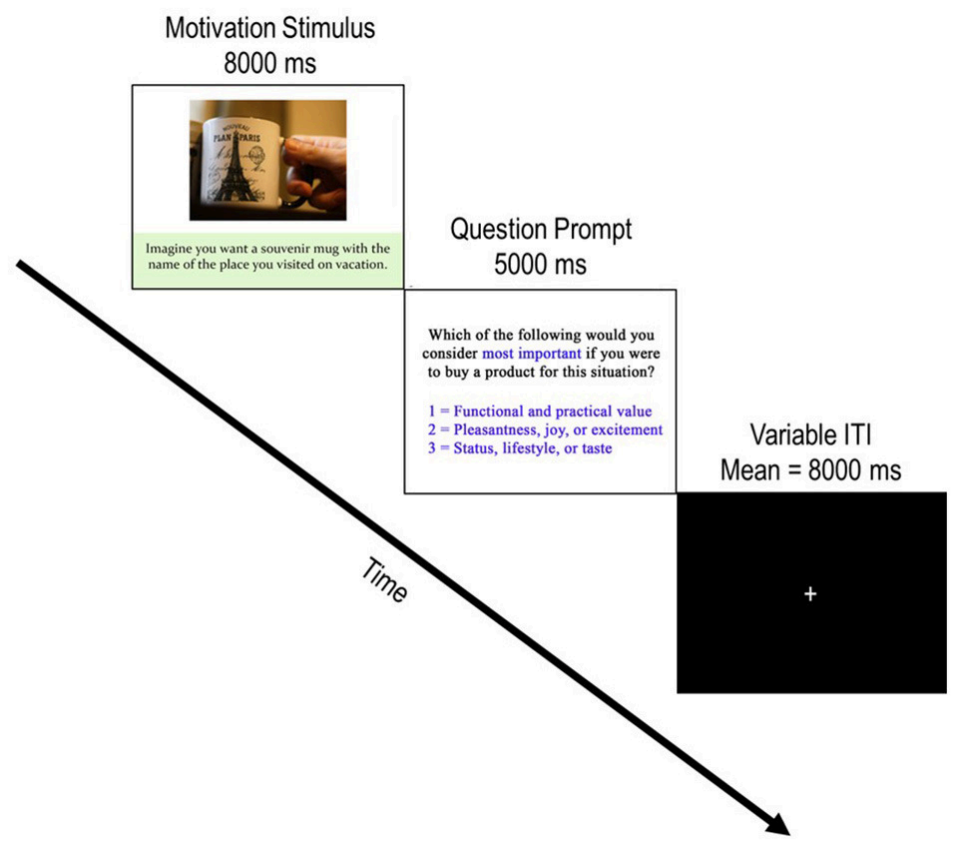
Depiction of a typical trial progression. Each trial begins with the presentation of a product stimulus accompanied by a buying motivation scenario. After the motivation stimulus was presented for 8,000 ms, the question prompt screen appeared for 5,000 ms and incited participants to report which buying motivation they considered by pressing 1 of the three possible responses. Following the question prompt, a jittered ITI with a central fixation appeared. Participants were instructed to focus on the fixation cross and awaited the next trial during these periods. Note that the example image is not part of the experimental stimulus material used in the current study. The copyright lies with the authors, and no permission was required for the reproduction of this image.

Each of the 126 buying scenario stimuli comprised a single trial of the task. Figure 2 depicts a typical trial progression (mean 13-s duration) which always began with the onset of a stimulus presentation (8-s duration), followed by a question prompt (5-s duration) and then progressed to a variable inter-trial interval (ITI; mean = 8-s) which contained a central fixation cross. The variable ITI served to jitter the onset of stimuli and conditions with TRs to better estimate the hemodynamic response function (HRF) which underlies motivational conditions. During the question prompt, participants indicated what they considered most important if they were to buy the product for the particular situation using three buttons from a standard, 4-button, MR-compatible button box (Current Designs, Philadelphia, PA). They pressed 1 for “the functional and practical value of the product” (functional motivation), 2 for “the pleasantness, joy, or excitement the product can offer” (experiential motivation), or 3 for “your status, lifestyle, or taste that the product shows” (symbolic motivation).

The task was divided into three blocks of 42 trials each. Optseq (http://surfer.nmr.mgh.harvard.edu/optseq/) was used to determine an ideal sequence of variable ITIs and trials corresponding to motivational conditions which maximized the variance of the predicted fMRI response and thereby minimized the overlap of HRFs during blocks of event-related fMRI designs. The inputs for Optseq were specified for the finite impulse response (FIN) window with no (0-s) minimum, a 10-s maximum, a minimum 1-s post-stimulus delay. Additionally, a variable ITI would last between 4-s and 10-s. The motivational conditions depicted by the visual/verbal scenarios were counterbalanced within each run to be presented an equal number of times.

### Experimental procedure

To ensure comprehension of the task requirements, all participants completed a practice version of the task prior to scanning using a standard PC, LCD monitor, and keyboard. The practice task was identical to the task implemented during scanning; however, only cars, pasta sauce, sunglasses, and tablet computers were presented with visual/verbal scenarios emphasizing either the functional, experiential, or symbolic values of each product. Following this practice version of the task, participants completed an additional practice version of the task using the same four practice stimulus items once inside the scanner, during a echo-planar imaging (EPI) BOLD acquisition sequence similar to the actual task scan. This additional practice task was conducted during MR scanning to ensure that participants were acclimated to the MR-compatible button box and rear-mounted projector screen, as well as the scanning environment during conditions identical to the actual imaging acquisition sequence. All experimental events during the practice task and actual task completed during scanning were presented using a standard PC, and MR-compatible rear-mounted projector screen and projector (Silent Vision, http://www.avotecinc.com/). Events were controlled and recorded using a custom program written with E-prime 2 software (http://www.pstnet.com/). However, only data from the actual tasks were analyzed and those from the practice task (both inside and outside the scanner) were not submitted to further analysis.

### Data collection

This study was carried out on a 7 Tesla (T) MAGNETOM scanner (Siemens Healthcare, Erlangen, Germany) using 32-channel head coil at the Auburn University MRI Research Center in Auburn, AL, USA. Prior studies have shown higher functional specificity using 7T vs. 3T in terms of percent signal change, mean *t*-values, number of supra-threshold voxels, and contrast to noise ratio (Beisteiner et al., 2011; Geißler et al., 2014). Thus, compared to using the same number of subjects at 3T, the likelihood of detecting true activations is better at 7T. Functional brain imaging data were acquired using a multiband echo-planar imaging sequence (Feinberg et al., 2010) with repetition time (TR) = 1-s, echo time (TE) = 20-ms, slice thickness = 2 mm, gap between slices = 3 mm, flip angle = 70°, in-plane resolution of 2 × 2 mm^2^, and multi-band factor of 2. Also, a high-resolution 3D MPRAGE sequence was used to collect T1-weighted structural data for anatomical localization for the fMRI data. Visual stimuli were presented to participants in three different runs per session, with each run lasting approximately 16 min. Runs were randomized for each participant, but were counterbalanced to consist of 42 trials with 14 trials for each of the three motivations, selected without replacement.

### fMRI data analysis

#### Preprocessing

All data preparation and preprocessing steps, as well as statistical analysis, were conducted with Statistical Parametric Mapping (http://www.fil.ion.ucl.ac.uk/spm/software/spm8/) under MATLAB environment. Standard image preprocessing was performed including realignment (motion correction), normalization, smoothing, and detrending. Motion correction was performed, to detect and correct for head movements, by spatial alignment of all volumes to the first volume by rigid body transformations. Translation and rotation parameters were inspected and never exceeded 1 mm or 1°, respectively. Then, we normalized MRI images into Montreal Neurological Institute (MNI) standard brain template space using nonlinear warping. All functional imaging data were spatially smoothed with 6 mm FWHM Gaussian kernel.

#### Statistical analysis

After preprocessing the raw data, BOLD fMRI data were analyzed in normalized space using a General Linear Model implemented in SPM8. The time course of brain activation was modeled with a boxcar function convolved with the canonical hemodynamic response function (HRF), including the time and dispersion derivative function allowing for variations in subject-subject level and voxel-voxel response. After obtaining the estimated β, voxel level inter-subjects and between-subjects linear contrasts were computed using different *t*-tests methods. The statistical threshold of significance was set at *p* < 0.05 (FDR corrected). Functional maps were overlaid on the MNI T1-weighted brain template.

In order to assess the neurofunctional correlates which underlie each of the three motivations for consumer decisions, task-related activations specific to the 8-s motivation stimulus presentation (see Figure 2) were compared between stimulus presentation durations for each trial type. Given the emotion-relevant information associated with experiential and symbolic motivations of consumer decisions, these motivation conditions should reflect relatively hot processing as compared to the relative cold processing in functional conditions. Accordingly, separate contrasts comparing functional and experiential motivation trials, and functional and symbolic trials were assessed. Because there was an expectation for considerable overlap between experiential and symbolic conditions, it was necessary to assess activations unique to experiential and symbolic motivations. A third contrast compared functional trials to both experiential and symbolic trials. Likewise, a fourth contrast compared experiential trials to both symbolic and functional trials. A final contrast assessed activations unique to symbolic motivation by comparing functional trials to both functional and experiential trials. All contrasts were computed using unpaired (pooling) *t*-tests. All comparisons were run as two-tailed tests; however, only contrasts that yielded significant differences in activation passing the FDR corrected threshold are reported.

## Results

### fMRI results

#### I. experiential motivation > functional motivation for display

**Figure 3:**
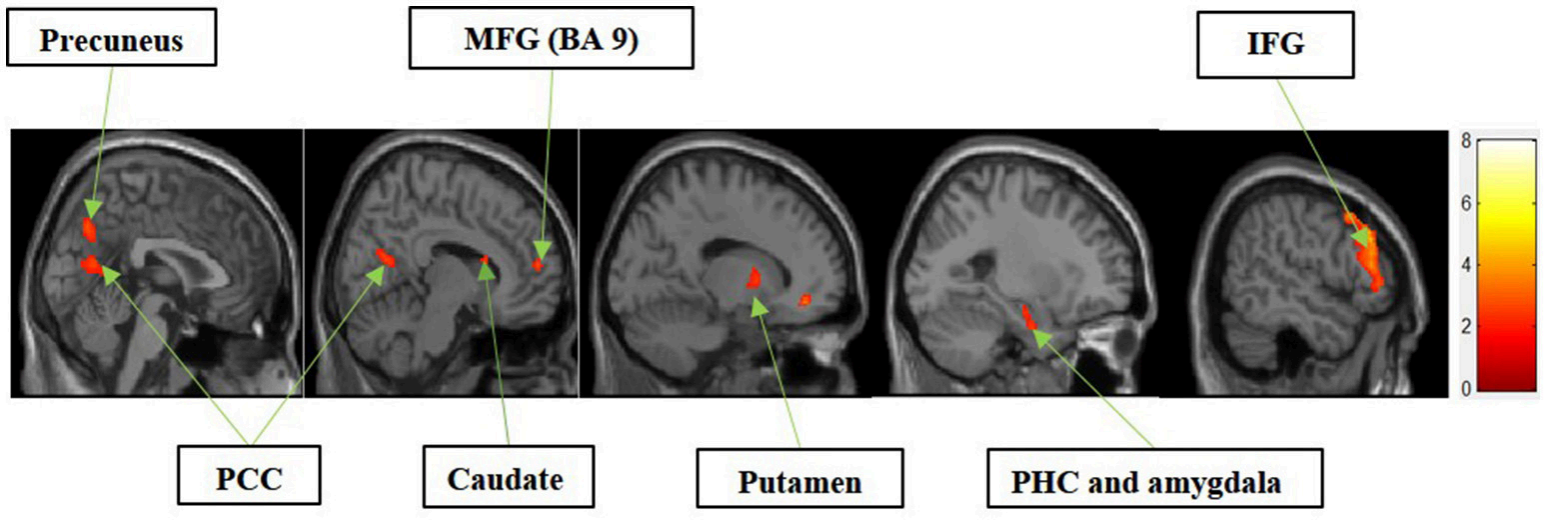
Experiential motivation > functional motivation. The color bar reflects *t*-values that resulted from the contrast. The precuneus, PCC, MFG (Brodmann area 9), caudate, putamen, parahippocampal gyrus, amygdala, and IFG showed significantly higher activation while processing products with experiential motivation as compared to functional motivation.

Figure 3 shows the results of *t*-tests for the contrast that compared experiential motivation to functional motivation for display condition. The results of this comparison yielded activations in the MFG, precuneus, PCC, caudate, putamen, parahippocampal cortex (PHC) and amygdala cluster, and inferior frontal gyrus (IFG).

#### II. symbolic motivation > functional motivation for display

**Figure 4:**
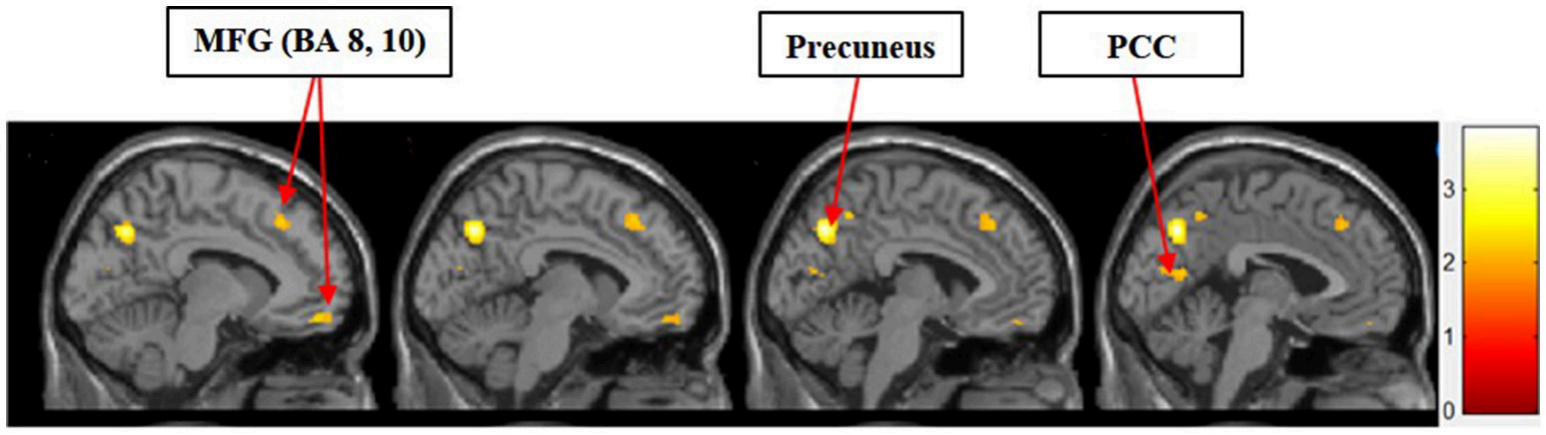
Symbolic motivation > functional motivation. The color bar reflects *t*-values that resulted from the contrast. The MFG (Brodmann areas 8, 10), precuneus, and PCC showed significantly higher activation while processing products with symbolic motivation as compared to functional motivation.

Figure 4 shows the results of *t*-tests for the contrast that compared symbolic motivation to functional motivation during stimulus display periods. The results of this comparison yielded activations in the MFG, precuneus, and PCC.

#### III. functional motivation > symbolic and experiential motivations for display

**Figure 5:**
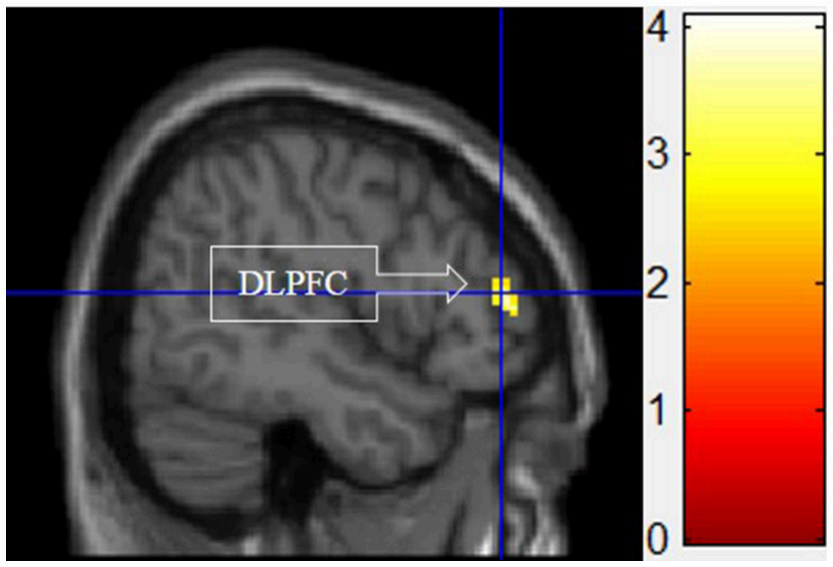
Functional motivation > symbolic and experiential motivations. The color bar reflects *t*-values that resulted from the contrast. The DLPFC showed significantly higher activation while processing products with functional motivation as compared to both symbolic and experiential motivations.

Figure 5 shows the results of *t*-tests for the contrast that compared the functional motivation to the experiential and symbolic motivations. Activation only in the DLPFC was significant.

#### IV. experiential motivation > functional and symbolic motivation for display

**Figure 6:**
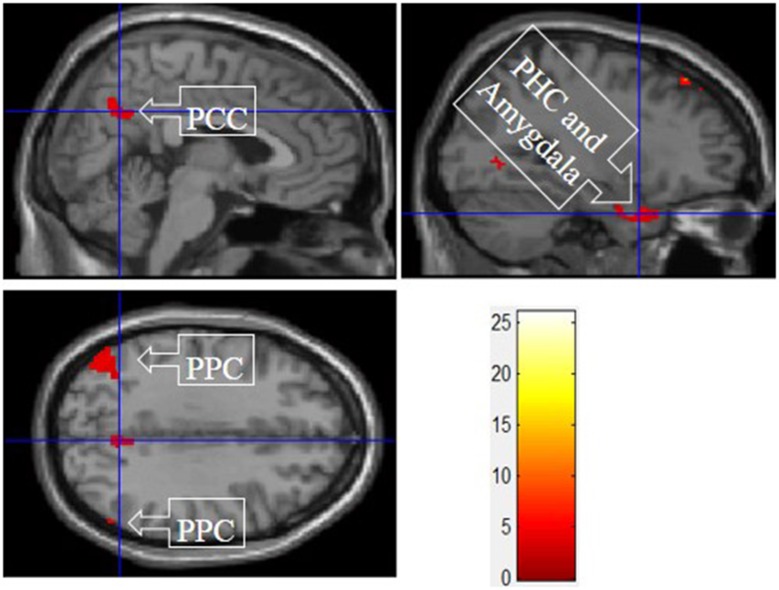
Experiential motivation > symbolic and functional motivations. The color bar reflects *t*-values that resulted from the contrast. The PPC, parahippocampal gyrus, and amygdala showed significantly higher activation while processing products with experiential motivation as compared to both symbolic and functional motivations.

Figure 6 shows the results of *t*-tests for the contrast that compared experiential motivation scenarios to the functional and symbolic motivations. Experiential motivation activated the PCC, and a cluster containing the PHC and amygdala.

#### V. symbolic motivation > functional and experiential motivations for display

**Figure 7:**
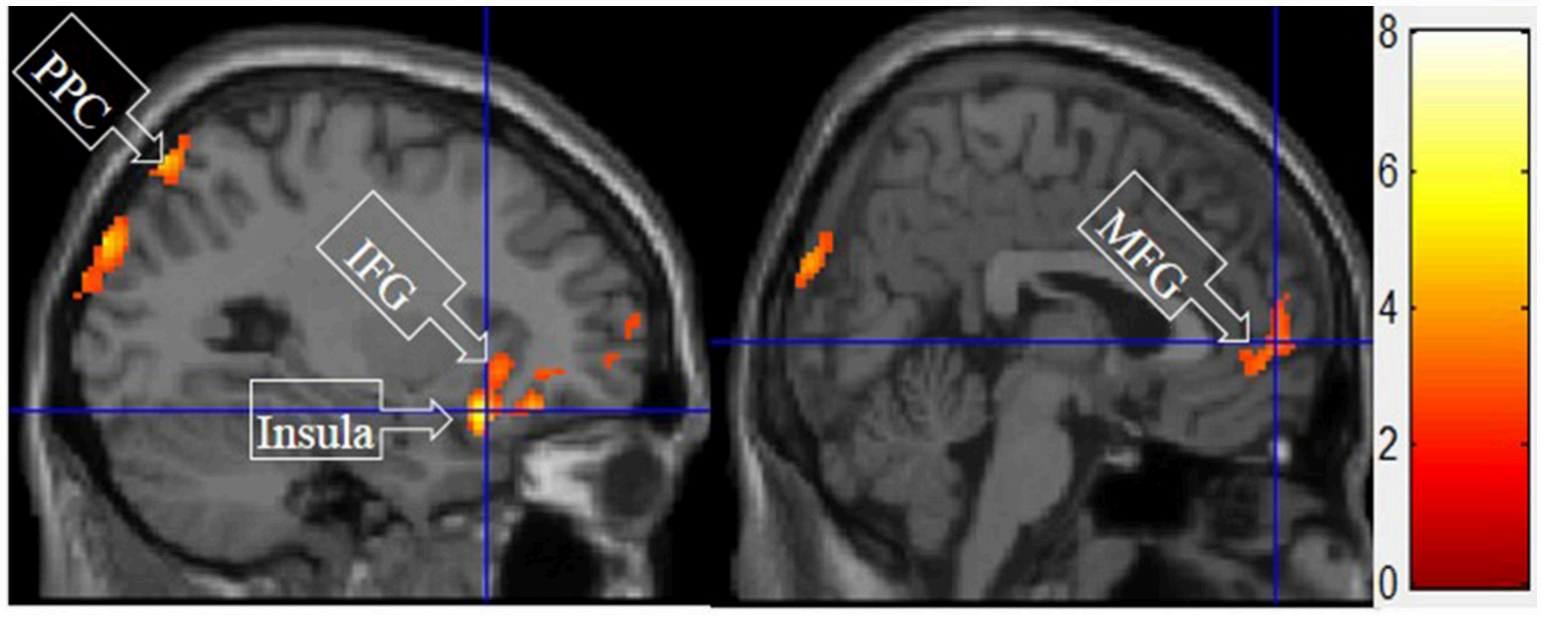
Symbolic motivation > experiential and functional motivations. The color bar reflects *t*-values that resulted from the contrast. The PPC, IFC, insula, and MFG showed significantly higher activation while processing products with symbolic motivation as compared to both experiential and functional motivations.

Figure 7 shows the results of *t*-tests for the contrast that compared symbolic motivation scenarios to the functional and experiential motivations. Symbolic motivation yielded significant activations in the MFG and IFG, as well as insula and posterior parietal cortex (PPC).

Detailed results of the group level voxel-wise analysis for contrasts of each of the three motivations, including peak MNI coordinates of the regions significantly activated (corrected *p* < 0.05), peak intensity (*t*-value), and cluster size (number of voxels) are reported in Table 2.

**Table 2:** Summary results from the functional contrasts of the motivation conditions.

**Condition**	**Region**	**Peak MNI coordinate (*x*, *y*, *z*)**	**Peak intensity (*t*-value)**	**Cluster size (voxel #)**
Experiential motivation > Functional motivation	Parahippocampal Gyrus	(−26, −6, −28)	2.42	72
	MFG	(16, 54, 10)	3.6	56
	Precuneus	(−4, −70, 40)	3.82	271
	Caudate	(12, 14, 16)	2.9	27
	PCC	(8, −64, 18)	2.8	126
	Putamen	(−16, 4, 2)	2.55	75
	IFG	(−56, 22, 8)	3.24	1017
Symbolic motivation > Functional motivation	MFG	(−10, 54, −14)	2.47	47
		(−8, 30, 44)	2.25	80
	Precuneus	(−2, −54, 50)	2.23	13
		(−2, −70, 42)	3.45	271
	PCC	(8, −64, 18)	2.8	126
Functional motivation > Experiential and Symbolic motivations	DLPFC	(48, 46, 10)	4.08	20
Experiential motivation > Functional and Symbolic motivation	PPC	(−46, −72, 24)	12.12	235
	PCC	(56, −66, 38)	4.75	32
		(6, −64, 36)	4.1	107
	Parahippocampal Gyrus	(32, 12, −32)	4.98	156
Symbolic motivation > Functional and Experiential motivation	Insula and IFG clusters	(36, 22, −20)	8.44	286
		(−30, 18, −16)	7.59	176
	PPC	(44, −84, 30)	9.17	283
	MFG	(14, 60, 2)	6.83	273

*This table reports the results of the group level voxel-wise analysis for contrasts of each of the three buying motivations. Columns indicate condition, region, peak MNI coordinates, peak intensity (t-value), and cluster size (number of voxels)*.

## Discussion

In the current study, we found that the MFG, precuneus, and PCC were differentially activated during both experiential and symbolic motivations (i.e., experiential motivation > functional motivation, see Figure 3 and symbolic motivation > functional motivation, see Figure 4). These findings indicate that unlike functional motivation, both experiential and symbolic motivations appear to be related to self-reflection and self-relevant processing. This conclusion is consistent with prior implications that the MFG is related to self-reflection processing (Schaefer and Rotte, 2007). The activation of the putamen and caudate (see Figure 3) shows experiential motivation is related to a reward mechanism. In addition, the amygdala activation (see Figure 3) demonstrates experiential motivation led to emotional processing, which we did not find for symbolic motivation.

As predicted, functional motivation demonstrated more activation in the DLPFC compared with other motivations (see Figure 5). The DLPFC has been widely studied and implicated in higher cognitive functions such as working memory, attention, decision-making, and executive control (O'Reilly, 2010). The current finding that functional motivation elicited the higher activation within the DLPFC is consistent with Keller's (1993) conceptual distinctions of this buying motivation, which is more associated with rational thoughts.

The consistent activation of the MFG, precuneus, and PCC suggested that there is no intrinsic difference between experiential motivation and symbolic motivation when contrasted with functional motivation. However, when experiential (or symbolic) motivation was contrasted against both functional and symbolic (or experiential) motivations, respectively, the results demonstrated that the PCC was only found to be activated under experiential motivation (see Figure 6) and the MFG was found to be exclusively activated under symbolic motivation (see Figure 7). The PCC and MFG are two regions that are shown to be involved in self-referential thought processing (Ochsner et al., 2004; Vogt and Laureys, 2005). However, the PCC is associated with inward self-reflection aimed at comparing oneself with others (Benoit et al., 2010), while the MFG is associated with outward self-reflection aimed to better understand others (Johnson et al., 2006). Thus, the current results suggest that both experiential and symbolic motivations are associated with self-reflection processes; however, they involve unique neural mechanisms associated with differing social judgment-making processes. Specifically, experiential motivation appears to be associated with the PCC and inward comparisons of oneself to others. Alternatively, symbolic motivation is associated with the MFG and outward comparisons of oneself to others. Additionally, activation in the PHC suggests that experiential motivation elicits episodic memory as well as contextual associations (Aminoff et al., 2013). The amygdala activation also demonstrates that experiential motivation likely led to higher emotional processing than other motivations, consistent with the hedonic nature of experiential motivation. Unlike experiential and functional motivations, the activation during symbolic motivation in the insular cortex could be due to interoceptive awareness (Critchley et al., 2004).

These findings are consistent with previous findings and our hypotheses, with the exception that there was no evidence of reward mechanisms being involved in the regulation of social relations such as dominance and social rank (Erk et al., 2002); instead, only experiential motivation was related to reward regions (i.e., caudate, putamen). These results are not without limitations. First, an experimenter error resulting in the loss of button responses during the task precludes strong evidence supporting notions that participants imagined the exact buying scenarios that they were instructed to maintain while viewing the product stimuli. However, it is unlikely that participants would disregard the instructions as they did not report any difficulty after the tasks were completed. A second limitation is the likelihood that imagining buying scenarios only simulates these otherwise intrinsic factors that arise from ecological consumer decisions. Although studies that examine such ecological buying scenarios would provide an alternative assessment to the current study, the level of control that provided the current results enabled maximal statistical power and spatial localization given the goals of the current study. A final limitation worth noting is the relatively small sample size in this study (*n* = 10). The current study serves as a preliminary neuroimaging assessment of neural correlates that vary between functional, experiential, and symbolic buying motivations. Accordingly, the findings of the current study serve as an initial assessment for the neural basis of consumer buying motivations and warrant further investigations with larger sample sizes to enhance the validity of the findings.

Notwithstanding these limitations, the current results shed light on the neural basis of consumer buying motivations. Importantly, we demonstrated functional dissociations consistent with reported models of distinct buying motivations (Keller, 1993). Within each condition of the current study, clusters of activation implicated neural correlates that were previously associated with specific types of information processing. The functional correlates implicated were largely in line with the understanding of the contributions of information processing that are said to underlie each motivational factor. Namely, functional buying motivation was associated with previously implicated cognitive control regions of the PFC (i.e., DLPFC), suggesting that such cognitive control might be suppressed under the experiential or symbolic buying conditions, potentially leading to less rational decision making.

An unexpected result was the common activation for both experiential > functional motivation and symbolic > functional motivation within the PCC and MFG. An interesting caveat to this common activation is that these buying motivations differed in activation when contrasted against every other condition. Specifically, the PCC was activated exclusively with the experiential motivation conditions, whereas the MFG was activated exclusively with the symbolic motivation condition. These findings may provide the neural basis of how self-referential valuations elicited by experiential and symbolic motivations are likely similar, yet different in subtle ways. Experiential motivation is elicited when consumers seek emotional benefits of consumption to fulfill hedonic needs (Keller, 1993). Thus, the high activation in the PCC as well as amygdala, putamen, and caudate with experiential motivation suggests that reward mechanism associated with episodic memory-based emotional arousal may guide internalizing self-reflection and comparisons under this motivation. On the other hand, symbolic buying motivation is elicited when consumers pursue social approval or self-enhancement in social settings (Keller, 1993). Thus, the high activation in the MFG with symbolic motivation suggests the instrumental, goal-directed nature of externalizing self-reflection and comparisons under this motivation. By examining the neural basis for different types of consumer buying motivations, this study contributes to enhancing the understanding of how consumer choices are made and why varying levels of self-control may be exerted under different buying motivational states.

## Ethics statement

This study was carried out in accordance with the recommendations of the Auburn University Institutional Review Board with written informed consent from all subjects. All subjects gave written informed consent in accordance with the Declaration of Helsinki. The protocol was approved by the Auburn University Institutional Review Board.

## Author contributions

AG collected the data. YW analyzed the data. AG and YW prepared the manuscript. WK, SB, JK, and GD provided the design of the study, supervision, and revisions on various drafts of the manuscript. AG revised the manuscript.

### Conflict of interest statement

The authors declare that the research was conducted in the absence of any commercial or financial relationships that could be construed as a potential conflict of interest.
