# Supplementary figures and images for: Neural Correlates of Consumer Buying Motivations: A 7T functional Magnetic Resonance Imaging (fMRI) Study
Source: Front Neurosci. 2017 Sep 14;11:512. doi: 10.3389/fnins.2017.00512 (PMC5603698; doi:10.3389/fnins.2017.00512)

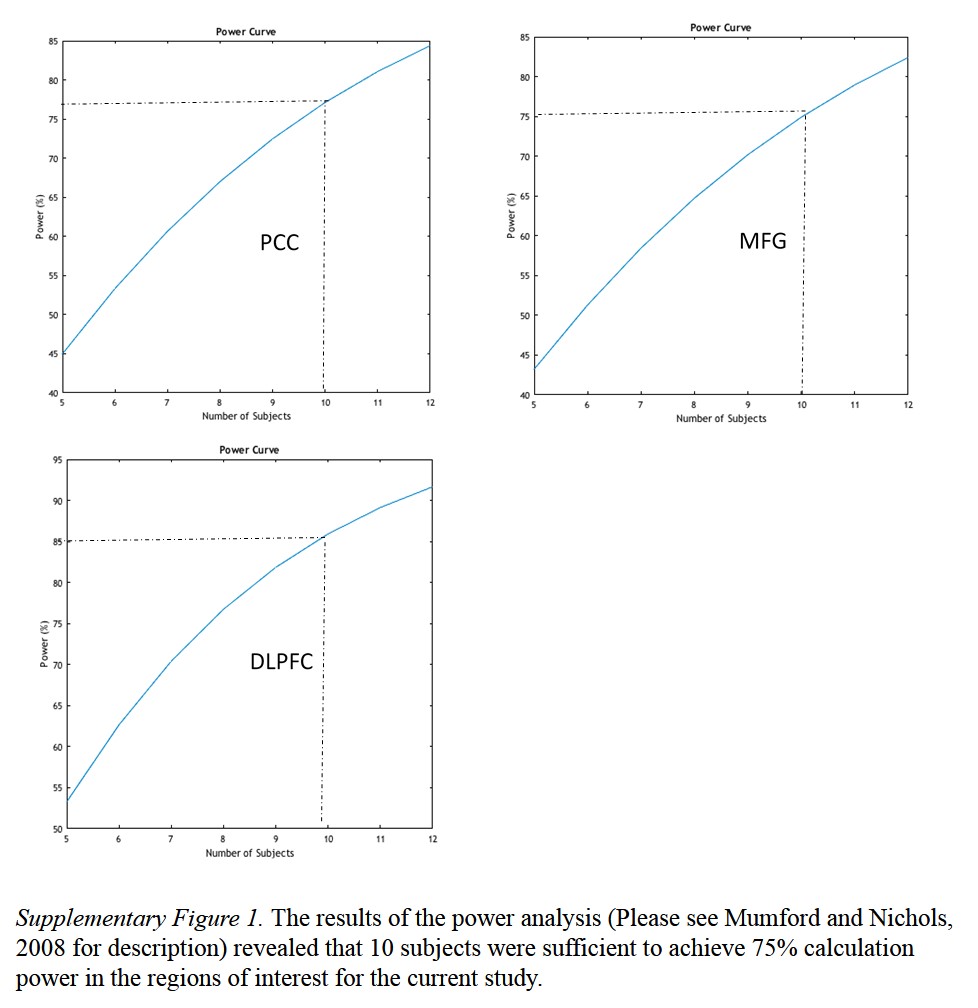

Supplement: Supplementary file 1 [file Image1.JPEG]
